# Experimental Study on Acoustic Emission Characteristics of Uniaxial Compression of MICP-Filled Sandstone

**DOI:** 10.3390/ma16093428

**Published:** 2023-04-27

**Authors:** Ling Fan, Chengbo Wang, Di Hu

**Affiliations:** School of Resources and Safety Engineering, Central South University, Changsha 410083, China; 205511022@csu.edu.cn (C.W.);

**Keywords:** fracture, MICP repair, uniaxial compression test, acoustic emission parameters, non-destructive

## Abstract

Rock masses are inherently heterogeneous, with numerous fractures that significantly affect their mechanical properties, fracture characteristics, and acoustic emission features due to the interactions between fractures or between fractures and the rock mass. Microbially induced calcite precipitation (MICP) technology, as an emerging non-destructive biological grouting reinforcement method, can repair fractured rock masses and alter their internal conditions. To investigate the mechanical properties, failure process evolution, and MICP repair effects of sandstone before and after repair, uniaxial compression tests were conducted on prefabricated, fractured (0.7–2.0 mm width) filled and unfilled rock samples, with acoustic emission monitoring throughout the process. Acoustic emission signal characteristics of the rock samples under stress were comparatively analyzed, determining the rock failure process and the microscopic failure types at compression-density stages, elastic stages, and destruction stages. The results show that the properties of the filled specimens improved, the failure process was mitigated, and the final failure stage was dominated by tension signals, accounting for over 60% of the total. The filling effect was better than 1.5–2.0 mm when the fracture width was 0.7–1.0 mm. The study deeply reveals the evolutionary process of compressive failure of the two types of rocks under different fracture widths, and by correlating the acoustic emission parameters with the stress–strain process, it provides a theoretical basis for repairing rock fractures using microbial engineering technology and offers experimental evidence and possible directions for the improvement and optimization of MICP technology.

## 1. Introduction

Fractured rock masses are prevalent in geological structures, and the complex interactions between the fractures themselves, as well as between fractures and the rock mass, make studying them a challenging task [1,2]. The expansion and connection of internal fractures can lead to instability of fractured rock masses, resulting in engineering accidents [1,2,3,4,5,6,7,8,9]. For example, in mine pillars, small fractures form seepage channels, leading to potential water inrush hazards; in rock slopes, rock mass damage caused by fractures can result in slope instability and failure; fractures developing and expanding on mountain tops and sides can lead to an increase in the frequency of landslides over several decades. As a result, the study of fractured rock masses has become a hot topic in the engineering field. Research by Xie H. et al. utilized true triaxial experiments to investigate the mechanical behavior and dynamic disaster behavior of fractured rock masses [10], Zhao Y.L. et al. employed uniaxial compression tests to study the crack initiation and stress–strain behavior of rock-like materials containing two fractures [11], and Liu Q.B. et al. used the discrete element method to investigate the strength and failure characteristics of double circular hole jointed rock masses under uniaxial compression [12]. Focusing on the study of fractured rock masses has significant implications and the potential to improve safety in engineering applications. As research in this field advances, attention has been given to the morphology of fractures. Wang J. et al. [13], Huang D. [14], Du K. [15], Xiang F. [16], and others have studied the influence of fracture angles on rocks and discovered the relationship between angles and failure modes. Wang G.L. et al. discovered the effects of four different types of fractures on rock properties [17]. Shen J.Y. [18], Xu Y. [19], and Ivan Gratchev [20] analyzed the rock damage process regarding fractures of different widths, discovering the relationship between fractures and rock formations and noting significant changes in fractures when they are smaller or larger than 1 mm. However, the aforementioned studies have presented significant variation in their crack-width measurements, thereby failing to comprehensively discuss the impact of crack width on the mechanical behavior of rocks within a suitable range.

In addition to the study of fracture properties, many scholars have made efforts to repair fractures. Injection is one of the effective ways to improve fractured rock formations. One effective approach to improving fractured rock masses is through various grouting methods. Among them, microbially induced calcite precipitation (MICP) has emerged as a notable non-destructive technique that has garnered attention. It is a widely occurring biogeochemical process in nature, also known as microbial mineralization, which can effectively reduce permeability and alter rock failure conditions [21,22]. MICP has several advantages, such as low slurry viscosity, low grouting pressure, and favorable ecological effects [23,24,25,26]. It has even been used for treating highly alkaline and heavy metal-containing waste [27], indicating its broad application prospects. However, previous research has focused on the concentration of bacterial liquids, culture liquids, temperature, and gelation degree [25,28,29], emphasizing the effect of grouting itself, while largely ignoring the significant limitations of fracture morphology on injection effects. In addition, the repair effect of MICP is greatly affected by the geometric morphology of fractures. Furthermore, evaluation indicators such as permeability, filling rate, and shear strength are commonly used to assess repair effects [29,30,31]. Scholars such as Peng S.Q. and Shen F. have studied the changes in permeability, strength, and other properties of fractures with different widths and roughness after filling. However, the mechanism of fractured rock formation damage is still unclear, and the changes in rock formation caused by repair effects, as well as their effects on the failure mode, have not been adequately considered. The changes in fractures caused by repair have also been neglected.

At present, with the advancement of experimental testing techniques and given the complex mechanical properties of fractured rock masses and the promising application prospects of MICP, research on MICP-filled rocks and the utilization of acoustic emission (AE) detection for studying rock failure processes has attracted widespread attention [32,33,34,35]. After filling, the rock transitions from a mineral structure to a composite of minerals and grouting products. Conventional fracture analysis and compressive strength assessment cannot fully describe the failure process and internal changes. Consequently, acoustic emission technology is often employed in post-repair rock experiments for detection and analysis, providing insight into the internal failure processes beyond surface damage and strength, and has been widely applied in the civil engineering and rock mechanics fields [17,36]. Acoustic emission signals released during the rock failure process contain important information about the rock failure mechanism and can be used to identify the failure types [32,37,38]. Basic acoustic emission parameters include AE counts, energy, average frequency, rise time, and amplitude. By analyzing these basic parameters, the AE counts, energy release levels, and failure types during the rock failure process can be studied. In this experiment, AE event rate, AE amplitude, AE count, AE energy, and RA–AF composite parameters were used to describe the acoustic emission characteristics of filled and unfilled specimens.

In this study, uniaxial compression acoustic emission tests [39,40] were conducted to investigate the failure modes and acoustic emission characteristics of MICP-filled rock samples with different prefabricated fracture widths. The AE ring count, AE energy, RA–AF composite parameters, and so on were used to investigate the filling effect and fracture mode of MICP-filled rock samples. The AE characteristics of filled and unfilled specimens were described, and changes before and after filling were obtained. In addition, the cumulative value was calculated to analyze the overall failure situation and cumulative effect, and the fracture mechanism of sandstone was explained. This study provides a theoretical basis for the use of microbial engineering technology to repair rock cracks and provides experimental evidence and possible directions for the improvement and optimization of MICP technology.

## 2. Materials and Methods

### 2.1. Sample Preparation

**Figure 1:**
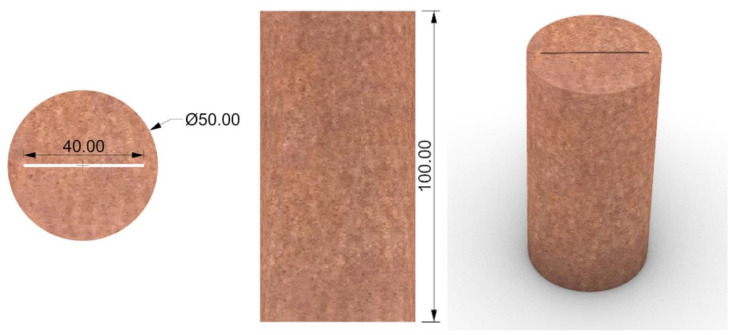
Dimensions of fractured sandstone samples.

**Figure 2:**
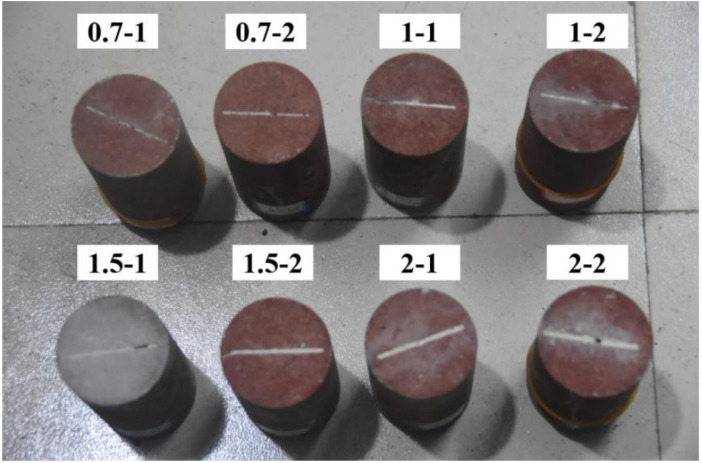
Grouted fractured red sandstone samples.

The red sandstone used in this experiment was prepared using a wire cutting device to ensure the bearing capacity of the specimen and obtain through straight cracks. Each sample was a cylinder (Φ 50 × 100 mm), with a diameter error of no more than 0.3 mm and an end-face non-parallelism error of no more than 0.03 mm. Four groups of samples were prepared with fracture widths of 0.7 mm, 1 mm, 1.5 mm, and 2 mm, with four samples in each group. Grouting was performed on samples 1 and 2 in each group. Before MICP grouting, the cracked rock samples were dried in a drying oven at 50 °C for 24 h, and the dry density of the rock was calculated after weighing. Then, the longitudinal wave velocity of the rock sample was measured using a YHB wave velocity meter. The rock sample was placed naturally in the laboratory (temperature 25 °C, humidity 40~45%), and the wave velocity probe was coated with a coupling agent. Measurements were taken every 2 h until the longitudinal wave velocity value of the sample stabilized. After MICP grouting, the grouted samples were placed in a drying oven at 50 °C for 24 h before being weighed again, and the longitudinal wave velocity was measured until it stabilized, as shown in Figure 1. The basic physical property measurements of the fractured sandstone before and after filling are presented in Table 1, and the treated sample is shown in Figure 2.

### 2.2. Analysis of Sandstone Composition

In this section, referring to the method of Liao C.Z. [41], the mineral composition of the red sandstone samples from Yunnan was analyzed using the X-ray diffraction (XRD) technique with an XRD-Advance D8 instrument from Bruker AXS Co., Ltd., Karlsruhe, Germany. The experimental procedure is described as follows:

1. Particle grinding: the intact sandstone was crushed and sieved using a mesh obtain red sandstone particles with a size of 0.08–0.075 mm, which were further ground to a particle size of 360 mesh.
2. Instrument calibration: the instrument was calibrated using the circulating water system until the water temperature reached 17–20 °C, and then the instrument was ready for testing.
3. Sample preparation: the red sandstone particles were pressed into pellets based on their characteristics and state.
4. Parameter setting: The sample was placed with its surface facing up at the detection position. The parameters were set as follows: normals were set, the start angle was set to 10°, and the stop angle was set to 90°; using a ceramic X-ray tube: the Cu target was set at 2.2 kW, 40 kV, 40 mA, with a step size of 0.02° and a counting time of 1 s. After the settings were completed, the testing began.

Based on the XRD diffraction spectrum obtained from the test results, the mineral composition of the red sandstone sample was mainly composed of quartz and feldspar, with quartz being the most abundant mineral, as shown in Table 1 and Table 2. The XRD experiment provided a certain experimental basis for the subsequent microscopic analysis of filling fracture rocks.

**Figure 3:**
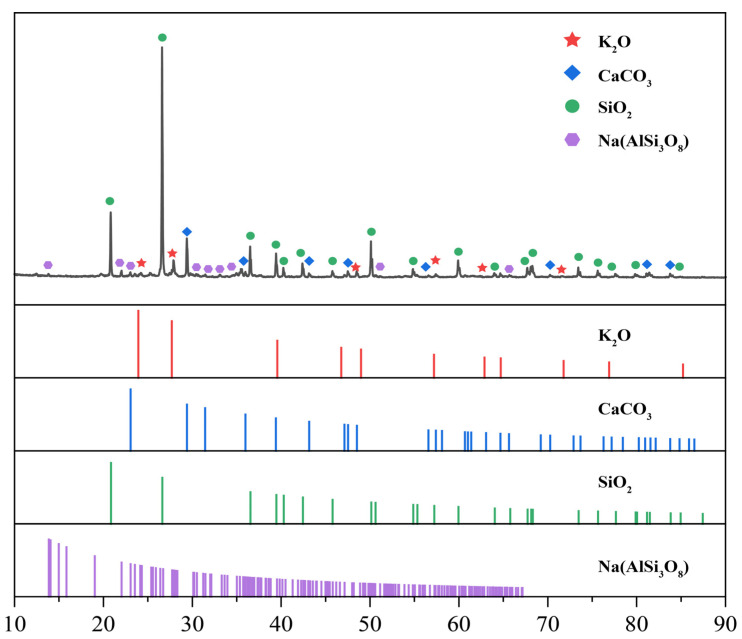
X-ray diffraction pattern of red sandstone.

Figure 3 shows the X-ray diffraction spectrum of the red sandstone sample. The spectrum was analyzed using Jade software to determine the mineral composition.

### 2.3. Experimental Scheme

The uniaxial compression test was carried out using an MTS815 rock triaxial test system from American MTS Systems Co., Ltd., (Eden Prairie, MN, USA), and the acoustic emission data were collected using the Micro-II Digital AE System hardware and its accompanying software. The experimental instruments and equipment used are shown in Figure 3. In order to obtain the complete stress–strain curve of the fractured sandstone before and after filling, all samples were loaded by displacement during the loading process, with a loading rate of 0.06 mm/min. The pre-stress was set to 0.1 kN, and the entire process involved continuously loading until the specimen failed. The axial and circumferential deformations of the specimen were recorded using an extensometer. The rock fracture acoustic emission signals were collected by a PCI-2-type acoustic emission receiver with a frequency range of 125–750 kHz and a center frequency of 140 kHz. The acoustic emission probe was fixed on the oil cylinder. To ensure sufficient coupling between the acoustic emission sensor and the oil cylinder and to reduce the attenuation of the acoustic emission signal at the interface between the sensor and the oil cylinder, a suitable amount of Vaseline was evenly applied to the contact surface between the acoustic emission probe and the oil cylinder. To eliminate the influence of environmental noise on the experimental results during the experiment, the threshold value for acoustic emission data acquisition was set at 40 dB. The acoustic emission waveform sampling rate was 1 megasample per second (MSPS). The stress-loading system and the acoustic emission-monitoring system were synchronized throughout the entire process of rock deformation and failure.

## 3. Filled Sandstone Wave Velocity Analysis

**Figure 4:**
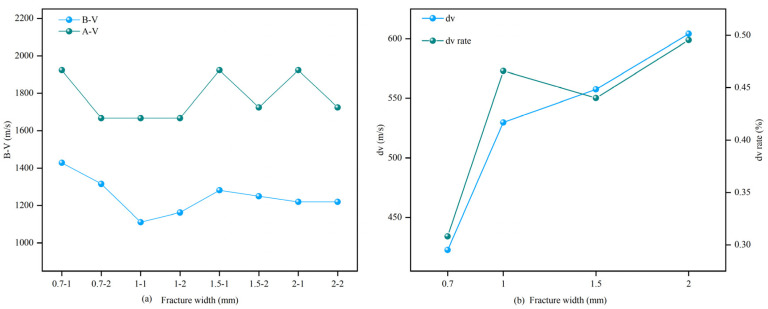
Variation of sandstone wave velocity before and after filling with different fracture widths: (**a**) wave velocity change, (**b**) wave velocity change rate.

Under the influence of long-term complex geological activities, underground rocks inherently possess a large number of joints and fractures, affecting the mechanical properties of the rock [42]. The longitudinal wave velocity of the rock is one of the important parameters reflecting the integrity and compactness of the rock itself [4]. To eliminate the impact of large initial fracture disparities on the experiment and improve the precision and reliability of the experiment, longitudinal wave velocity analysis was conducted on the sandstone, and the variation law of the longitudinal wave velocity of the sandstone with different fracture widths before and after MICP filling was obtained, as shown in Figure 4. As can be seen from Figure 4a, the longitudinal wave velocities of the samples with different fracture widths before filling are close, indicating that the sample properties are similar, providing reliability for the subsequent uniaxial compression test data. The post-filling wave velocity (vb) is approximately 30–50% higher than the pre-filling wave velocity (va), indicating that the rock fractures have been effectively filled and the fracture structure has been improved. Figure 4b shows the change in wave velocity (dv) and the change rate (dv-rate) before and after filling, and the wave velocity changes and change rates corresponding to different fracture widths are calculated from the average wave velocities of the two samples before and after filling. As the fracture width increases, the change in the wave velocity before and after filling the samples ranges from 400–650 m/s. When the fracture width is 2 mm, the post-filling wave velocity reaches 1924 m/s, and the change in the wave velocity and the change rate before and after filling are the largest. This result is consistent with the change in filling conditions and filling quality.

## 4. Experimental Results Analysis

**Figure 5:**
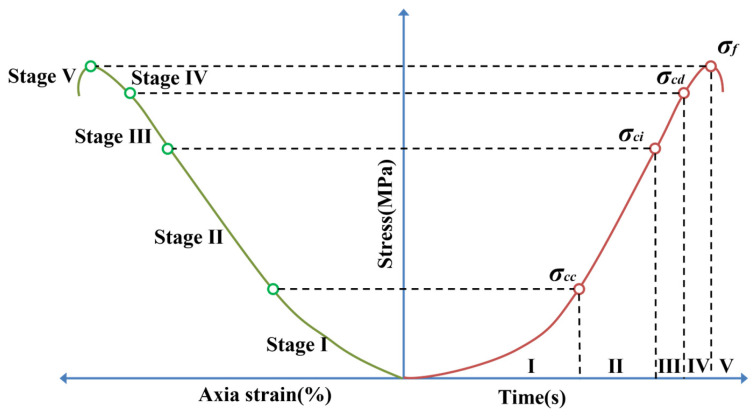
Stress–strain curve stage division.

Based on the indoor uniaxial compression acoustic emission tests, the stress–strain curves of red sandstone specimens with different fracture widths, both with and without MICP filling, were divided into five stages, as shown in Figure 5. The experimental results were analyzed in terms of acoustic emission event rate, amplitude characteristics, count, and energy.

### 4.1. Acoustic Emission Event Rate

(1) 0.7 and 1.0 mm

**Figure 6:**
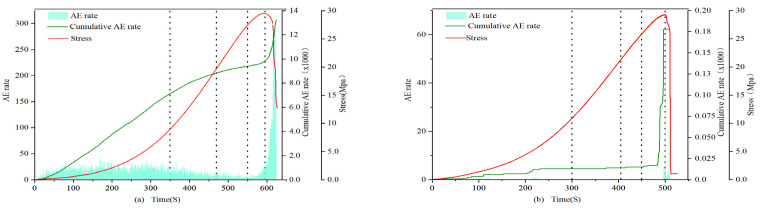
Event rate of 1.0 mm filled sample (**a**) 1-1 and unfilled sample (**b**) 1-3.

In the 0.7 mm and 1.0 mm groups, the acoustic emission event rate of the filled samples was much higher than that of the unfilled samples during almost the entire stage, with the maximum cumulative difference reaching 3 orders of magnitude. Among them, the difference between the compaction stage and the elastic stage is the most significant. From the perspective of the cumulative event rate, the curve of the filled samples is relatively full, and the growth trend is stable (as shown in Figure 6). It is higher in the first and second stages, declines in the third and fourth stages, and begins to rise again at the end of the fourth stage, consistent with the failure pattern. However, the curve of the unfilled samples shows a stronger surge, being at a lower level in the first three stages and then sharply increasing to a higher level at the end of the fourth stage, exhibiting an “L”-type characteristic. This indicates that, in the early stage, the filling material experienced the compaction process of the unfilled sandstone sample fractures, and the MICP filler effectively cemented the internal fractures of the rock. However, due to the relatively low strength of the filler, more acoustic emission events were generated. Through event rate analysis, we can conclude that the internal filling samples mainly involve fracture compaction expansion, and the filling weakens the trend of brittle failure in the rock.

(2) 1.5 mm

**Figure 7:**
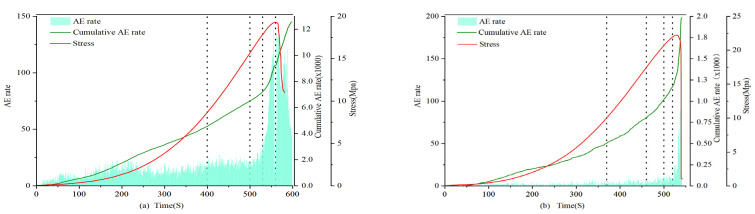
Event rate of 1.5 mm filled sample (**a**) 1.5-1 and unfilled sample (**b**) 1.5-3.

The trend of the filled samples is similar to that of the previous group, but the growth rate of events is more stable (as shown in Figure 7). The unfilled samples have more acoustic emission signals than the unfilled samples in the previous group, and the arrival of large-scale acoustic emission events is about 200 s earlier than in the previous group. The distribution is broader, the growth is stable, and the “L” type surge phenomenon is not obvious.

(3) 2 mm

**Figure 8:**
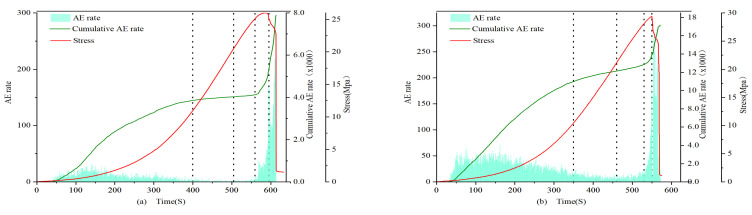
Event rate of 2.0 mm filled sample (**a**) 2.0-1 and unfilled sample (**b**) 2.0-3.

The total number of acoustic emission events in the two types of samples is close, and the filled samples have a more stable growth rate and a more uniform event distribution. This indicates that the filler has alleviated the damage process to some extent, but the repair effect is limited (as shown in Figure 8).

Among all the filled samples, the difference in the number of characteristics of uniaxial compression fracture before and after filling of 0.7 mm and 1 mm fracture sandstone is the most significant. Compared with the unfilled samples, the 0.7 mm and 1.0 mm groups increased nearly 100 times. Because the bonding strength of the calcium carbonate precipitation filling is relatively low compared to that of the rock, the bonded calcium carbonate begins to gradually break and produce acoustic emission phenomena as stress loading commences.

The difference in acoustic emission events between 1.5 mm and 2 mm samples is not pronounced, which can be attributed to their smaller calcium carbonate precipitation filling rates compared to those of the 0.7 mm and 1 mm samples. In all unfilled samples, there are fewer events during the initial loading stage, with a significant increase observed in the second and third stages. This increase accelerates as fractures develop, reaching its maximum value in the fifth stage. The fundamental reason is that the binding between red sandstone rock particles is not tight enough. Therefore, in the initial loading stage, when the local stress on the rock exceeds the particle bonding strength of the location, micro-cracks are generated, forming acoustic emission events. These micro-cracks expand into macro-cracks when the stress reaches the peak strength of the rock, generating a large number of acoustic emission events.

Overall, as the fracture width increases, the acoustic emission event rates of the two types of samples gradually approach each other. Unfilled samples have higher brittleness and more sudden failure, while filled samples have lower brittleness and more stable failure. As the fracture width increases, the filling effect changes from good to unstable. Considering the quality of the generated calcium carbonate precipitation, the reason is that the calcium carbonate inside the sample is insufficient to completely fill the fractures, resulting in fewer acoustic emission events, and the fracture width is close to the maximum width of the effective filling of MICP.

### 4.2. Acoustic Emission Amplitude Characteristics

(1) In the 0.7 and 1.0 mm group, the filled specimens exhibit an overall satisfactory and uniform distribution of acoustic emission signals without abrupt changes, showing an overall trend of initial increase, followed by a decrease, and then another increase, consistent with the compression experiment process. In contrast, the unfilled specimens have fewer acoustic emission signals, mainly concentrated in the range of 40–55 dB, with almost no signals above 60 dB. In the first three stages, the distribution of acoustic emission signals in the unfilled specimens is relatively uniform and low, while in the fourth and fifth stages, it is more concentrated.
(2) In the 1.5 mm group, the difference between the filled and unfilled specimens is reduced, with the distribution pattern of the unfilled specimens being similar to that of the previous group, but with the total number being approximately 10 times that of the previous group. The distribution density of AE amplitudes in the unfilled specimens is significantly smaller than that of the filled specimens in different stages. Particularly in the compaction stage, acoustic emission events occur sporadically and randomly, with generally low AE amplitudes, mostly below 50 dB. As in the previous group, with the continuous expansion of microcracks approaching the critical state, the accumulated elastic energy is instantaneously released due to the brittle failure of the sandstone specimens, resulting in a higher density of acoustic emission amplitudes for both filled and unfilled specimens. This indicates that multiple acoustic emission signals occurred in a short period, with the amplitude rapidly increasing to the peak, but lasted for only a short time. Meanwhile, as the crack width increases, the density of the AE amplitude distribution also increases.
(3) In the 2.0 mm group, the distribution of acoustic emission signals in both filled and unfilled specimens is similar, with similar patterns in each stage.

**Figure 9:**
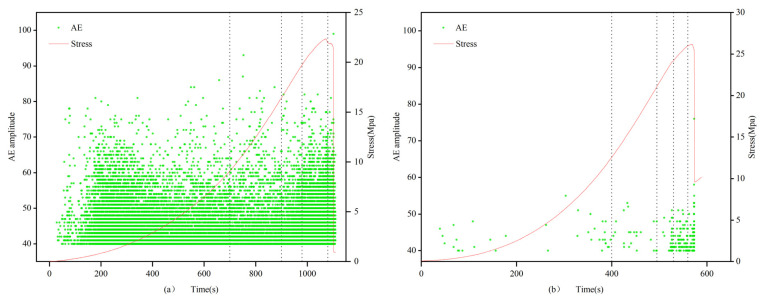
Amplitude characteristics of filled (**a**) 0.7-1 and unfilled (**b**) 1-3 specimens.

**Figure 10:**
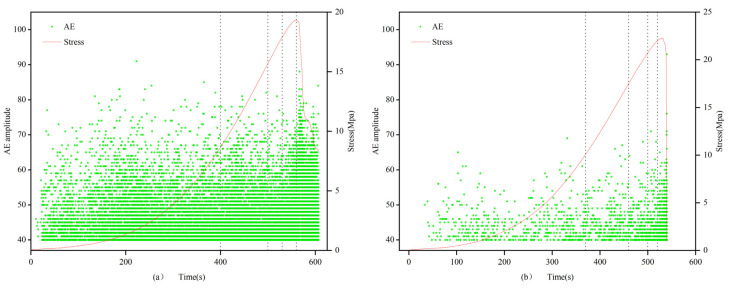
Amplitude characteristics of filled (**a**) 1.5-1 and unfilled (**b**) 1.5-3 specimens.

**Figure 11:**
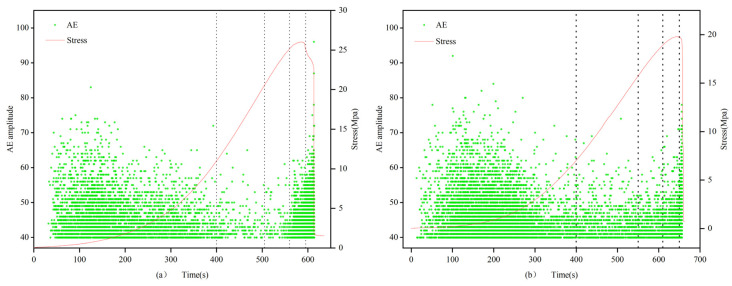
Amplitude characteristics of filled (**a**) 2-1 and unfilled (**b**) 2-3 specimens.

Figure 9, Figure 10 and Figure 11 show the curves of axial stress and acoustic emission (AE) amplitude changes with time under uniaxial compression conditions, where the left side represents the filled specimens, and the right side represents the unfilled specimens. The change patterns of AE amplitude in the filled specimens are consistent with the stress–time curve. In the first stage, due to the presence of natural fractures within the specimen, the acoustic emission activity significantly increases during the compaction process, resulting in a larger density of AE amplitude distribution. In the second stage, there are fewer internal acoustic emission activities in the specimen, showing a scattered distribution and sparse density. In the third and fourth stages, internal microcracks in the rock develop stably, crack propagation occurs, and acoustic emission activity intensifies, leading to a gradual increase in the density of AE amplitude distribution and a denser distribution. In the fifth stage, as strain energy accumulates, internal microcracks expand into large through-cracks, further intensifying the acoustic emission activity and reaching the highest degree of AE amplitude distribution density. Ultimately, the AE amplitude of the filled fracture specimen presents a trend of initial increase, followed by a decrease and then another increase over time.

The amplitude in the elastic stage of the filled specimen is higher, which is related to the difference in the elastic stage between the filling material and the rock material due to the strength difference. In the second and third stages, when there are fewer acoustic emission events in the elastic stage of the rock material, there are more acoustic emission events in the filling material. This suggests that the filling material has a significant impact on the acoustic emission activity of the specimen, further reflecting the interaction between the filling material and the rock material. This finding provides an important basis for studying the dynamic response characteristics of filled fracture specimens.

### 4.3. Acoustic Emission Count Characteristics

(1) 0.7 and 1.0 mm:

The count and cumulative value of filled and unfilled specimens show significant differences at each stage of rock stress. In the first stage, the amplitude and growth rate of the filled fracture specimens are larger, and the cumulative count of filled specimens increases from 0 to 3.0 × 10^5^, while the unfilled specimens only grow to 2.8 × 10^2^, with a difference reaching 3 orders of magnitude. In the second stage, the growth rates of the filled and unfilled fracture specimens are similar. From the third stage to the fourth stage, the cumulative count growth rate of the filled specimens accelerates, while the growth rate of the unfilled fracture specimens accelerates starting from the third stage, reaching its maximum in the fourth stage at approximately 4.8 × 10^3^. Throughout the entire stress process, the cumulative AE count curve of the unfilled specimens shows an “L”-shaped change, with a sharp increase in the fourth stage; the filled specimens exhibit an “S”-shaped change, with stable growth and peak values far higher than the former group. These results indicate that the filling material has a significant impact on the AE count characteristics of the specimens, revealing the differences in dynamic response characteristics between filled and unfilled specimens at different stress stages.

(2) 1.5 mm:

In the first stage, the number of AE counts greater than 600 in filled fracture specimens is higher than that in unfilled specimens, and the cumulative value of AE counts is also larger, with an extremely fast growth rate. From the second stage to the third stage, the growth rate of filled specimens is still higher than that of unfilled specimens, but the difference decreases; the filled specimens exhibit stable growth in the fourth stage. In the fifth stage, both types of specimens show a steep increase in the cumulative AE count, with the cumulative AE count of unfilled fracture specimens reaching 10^5^ and that of filled specimens reaching 10^6^. By comparing the calcium carbonate generation of specimens 1.5-1 and 1.5-2, it is likely that the significant difference in AE cumulative counts is due to the smaller calcium carbonate filling rate in specimen 1.5-2, resulting in the through-cracks not being filled properly and forming fractures of different shapes. Considering the evolution characteristics of AE counts throughout the entire uniaxial compression process, it is found that both well-filled specimens and unfilled specimens show “L”-shaped changes in their cumulative AE count curves.

(3) 2 mm:

In the first stage, the cumulative AE counts of both filled and unfilled specimens increase initially and then remain essentially unchanged, but the cumulative count of unfilled specimens is twice that of filled specimens. In the second and third stages, the cumulative counts of filled and unfilled specimens remain essentially unchanged. In the fourth stage, both types of specimens show a sharp increase in their cumulative count, and the final cumulative counts of the two are not significantly different. Considering the change characteristics of the four stages, both filled and unfilled fracture specimens exhibit “S”-shaped changes.

Apart from the 2.0 mm specimens, the count characteristics of specimens of other fracture widths show that the counts of filled specimens are generally greater than those of unfilled specimens. In the first four stages, the growth rate of the AE count cumulative value curve is higher than that of the unfilled specimens. Specifically, in the first stage, the growth range of filled specimens is larger, and the density is higher than that of the unfilled specimens. Starting from the second stage, filled specimens still maintain an advantage, but the gap narrows. In the third and fourth stages, both types of specimens have relatively large counts, and the gap continues to narrow. In the fifth stage, both types of specimens experience a sharp increase, and growth is evident, indicating that with the rapid expansion of internal fractures in the specimen, the rock sample approaches failure, and AE activity becomes more intense.

**Figure 12:**
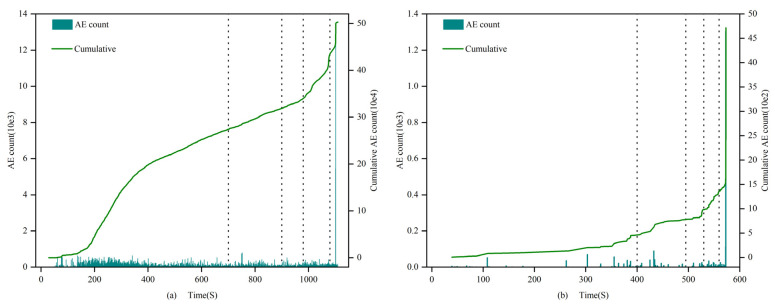
AE count of 0.7 and 1.0 mm filled specimens (**a**) 0.7-1 and unfilled specimens (**b**) 1-3.

**Figure 13:**
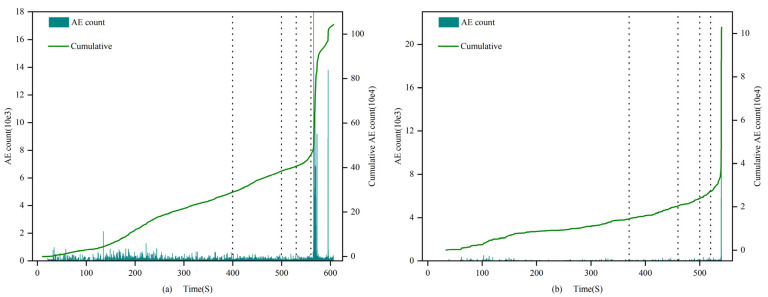
AE count of 1.5 mm filled specimens (**a**) 1.5-1 and unfilled specimens (**b**) 1.5-3.

**Figure 14:**
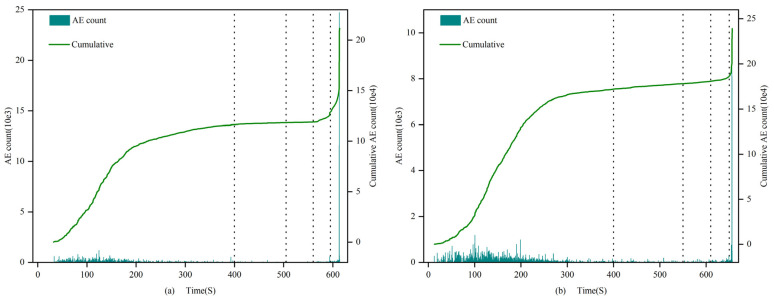
AE count of 2.0 mm filled specimens (**a**) 2-1 and unfilled specimens (**b**) 2-3.

The AE count characteristics of the specimens can be seen in Figure 12, Figure 13 and Figure 14, For 2 mm fracture-width specimens, the AE count cumulative value curve exhibits “S”-shaped change characteristics, suggesting that before sample failure, specimens with larger fracture widths under uniaxial compression stress generate higher-intensity acoustic emission events. Apart from the 2 mm specimens, all unfilled fracture specimens’ AE count cumulative value curves show “L”-shaped changes. This indicates that when the rock fracture width is 2 mm, the cumulative value change pattern of AE counts before and after filling is similar, and the MICP filling does not significantly alter the acoustic emission characteristics of the rock.

### 4.4. Acoustic Emission Energy Characteristics Analysis

(1) 0.7 and 1 mm

The AE energies of both types of specimens exhibit significant differences, with those after filling being significantly greater than those before filling. In the first stage, the energy density of the filled specimens is larger, showing a step-like development, while the energy of the unfilled specimens is smaller or even zero. In the second and third stages, the cumulative value of AE energy for filled specimens continues to rise, while the cumulative value of AE energy for unfilled specimens remains essentially unchanged. In the fourth stage, the AE signal of the filled specimens surges, but the energy value concentration is lower than that of the first stage, while the unfilled specimens sporadically exhibit AE signals. In the fifth stage, the post-peak fracture stage, the AE energy density of all specimens increases sharply. Similar to the evolution of AE counts, filled 0.7 mm fracture specimens exhibit an “S”-shaped change, while unfilled specimens exhibit an “L”-shaped change. The cumulative values of AE energy for filled specimens 0.7-1 and 0.7-2 reach 1.6 × 10^5^ and 1.4 × 10^5^, respectively, which are 3 orders of magnitude higher than those of the unfilled specimens 0.7-3 and 1-3 at 8 × 10^2^.

(2) 1.5 mm

In the first stage, filled specimens exhibit a large amount of energy release, showing an “S”-shaped change, while unfilled specimens still exhibit an “L”-shaped change, but the difference between the two is reduced, and the increase of the unfilled specimens is not as great as that of the previous group of unfilled specimens, similar to the change pattern of AE counts.

(3) 2 mm

In the first stage, the cumulative values of AE energy for both types of specimens develop in a climbing slope style. In the second and third stages, the cumulative value of AE energy is relatively stable, with no dramatic fluctuations, and both types of specimens show a consistent, slow increase in AE energy change patterns. In the fourth and fifth stages, energy fluctuations change most significantly, with rock instability and failure occurring, and the cumulative energy reaches its maximum at the peak. The cumulative AE energy values between specimens do not differ significantly. From the perspective of fracture width and acoustic emission (AE) energy, in the first four stages, as the fracture width increases, the AE energy density of the non-filled samples becomes larger. This indicates that the average energy released by micro-cracks during the compression, development, and expansion stages gradually increases, intensifying the damage to the rock. In the fifth stage, all samples exhibit a sharp increase in AE energy before failure, which corresponds to the post-failure stage of the rock. At this point, under the influence of axial stress, a large number of micro-cracks within the samples rapidly develop and expand, eventually forming large through-cracks, and a substantial amount of energy is quickly released.

**Figure 15:**
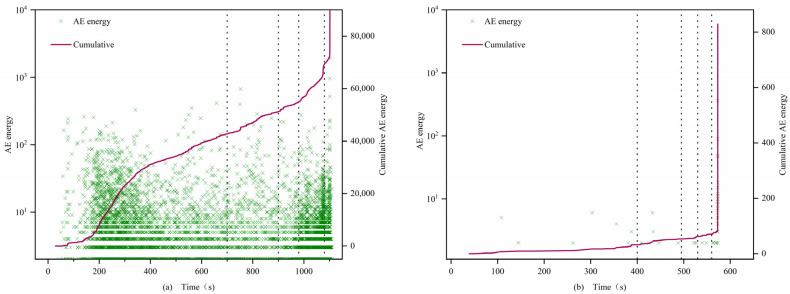
Energy characteristics of 0.7 and 1.0 mm filled (**a**) 0.7-1 and unfilled (**b**) 1-3 samples.

**Figure 16:**
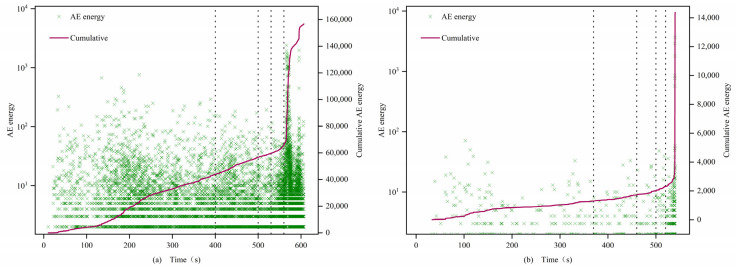
Energy characteristics of 1.5 mm filled (**a**) 1.5-1 and unfilled (**b**) 1.5-3 samples.

**Figure 17:**
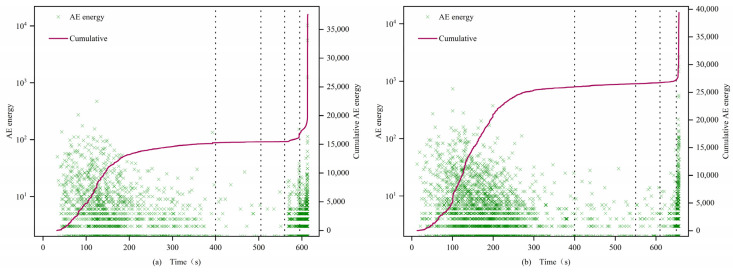
Energy characteristics of 2.0 mm filled (**a**) 2-1 and unfilled (**b**) 2-3 samples.

Figure 15, Figure 16 and Figure 17 illustrate the characteristics of the AE energy and cumulative values of the specimens. The analysis results show that the AE energies of 0.7 mm, 1 mm, and 2 mm samples are consistent with the evolution characteristics of AE event rate and AE count accumulation, indicating a strong correlation between the acoustic emission characteristic parameters of filled and unfilled samples at these fracture widths. Moreover, there is a significant difference in the maximum energy released at the moment of failure between filled and unfilled samples at 0.7 mm, 1 mm, and 1.5 mm, with a general difference of 2 orders of magnitude and the highest difference reaching 3 orders of magnitude. This demonstrates that MICP filling can affect the maximum energy released during rock failure at these fracture widths, reflecting the obvious filling effect.

Through the analysis of acoustic emission parameters, it has been observed that the number of acoustic emission events in the initial loading stage of unfilled fractured sandstone samples is relatively low for all fracture widths, with parameter values also being lower. These values significantly increase from the elastic stage to the micro-crack crack propagation process stage and reach their maximum in the post-failure stage of the rock. The difference in the number and parameter values of acoustic emission events before and after uniaxial compression fracture of 0.7 mm and 1 mm fractured sandstone samples is quite significant, with the difference in order of magnitude ranging from 10–1000. This is because the filling effect for 0.7 mm and 1.0 mm fractured rocks is good, altering the internal fracture conditions of the rock and changing the rock failure mode. As the fracture width increases beyond the effective repair range, the grouting adhesion effect deteriorates, reducing the fracture width but not changing the failure mode, resulting in minimal changes in acoustic emission phenomena. The study shows that as the fracture width increases from 1.5 mm to 2.0 mm, the MICP filling effect gradually weakens. When the fracture width reaches 2.0 mm, the filling effect is no longer significant.

These analytical results provide us with an in-depth understanding of the impact of MICP filling on the mechanical behavior and failure process of rocks at different fracture widths, which is beneficial for achieving more optimized rock reinforcement and stabilization measures in engineering applications.

## 5. Analysis of Sandstone Failure Modes Based on Acoustic Emission

### 5.1. Acoustic Emission RA–AF Analysis

Both theoretical research and experiments have shown that micro-cracks in the rock failure process mainly include two forms: shear cracks and tensile cracks, which determine the failure mode of the rock. Although the analysis results of the abovementioned AE data and cumulative values can reflect the overall evolution characteristics of micro-cracks in the rock failure process to a certain extent, they cannot determine the failure characteristics of the rock under compressive load. Therefore, this study adopts the acoustic emission waveform parameter index RA–AF to reveal the rock failure mechanism. The calculation methods of RA and AF are as follows:

(1) $$\mathrm{AF}=\frac{\mathrm{AE}\;\mathrm{Count}}{\mathrm{Duration}\;\left(s\right)}$$

(2) $$\mathrm{RA}=\frac{\mathrm{Rising}\;\mathrm{Time}\left(\mathrm{ms}\right)}{\mathrm{Amplitude}\left(V\right)}$$

**Figure 18:**
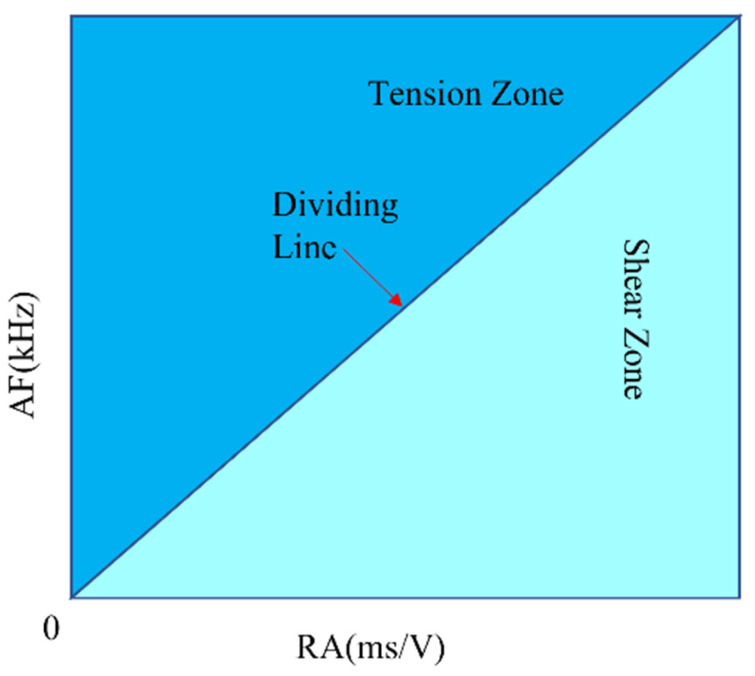
RA–AF schematic diagram.

RA and AF are derived from a large number of acoustic emission waveform signal analyses and summarizations. For tensile cracks, due to the instantaneous nature of tensile failure, elastic energy is instantaneously released, resulting in larger amplitude, more counts, and shorter duration. This is reflected in the results of the above two parameters as low RA and high AF, while shear cracks are the opposite. Therefore, the ratio of RA to AF can distinguish the type of crack to a certain extent. In the parameter analysis method of JCMS-III B5706, it is believed that in an RA–AF scatter plot, the ratio of AF to RA can be compared with a diagonal line with a specific slope as the criterion for distinguishing between tensile and shear cracks. As shown in Figure 18, by drawing the RA–AF scatter plot, points falling on the upper end of the dividing line represent tensile signals, resulting in tensile cracks; points falling on the lower end represent shear cracks. Numerous studies have shown that it is feasible to use a diagonal line with a specific slope to distinguish between tensile and shear cracks. Through this method, this study aims to provide a more accurate way to distinguish between tensile and shear cracks during the rock fracture process, thereby enabling better understanding of the rock fracture mechanism and its mechanical behavior.

**Figure 19:**
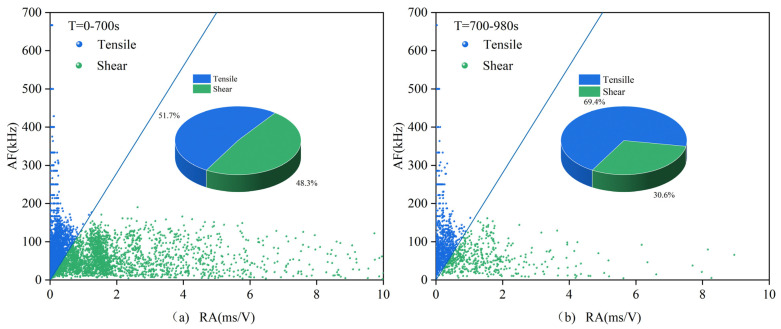
RA–AF scatter plot for the filled sample 0.7-1.

**Figure 20:**
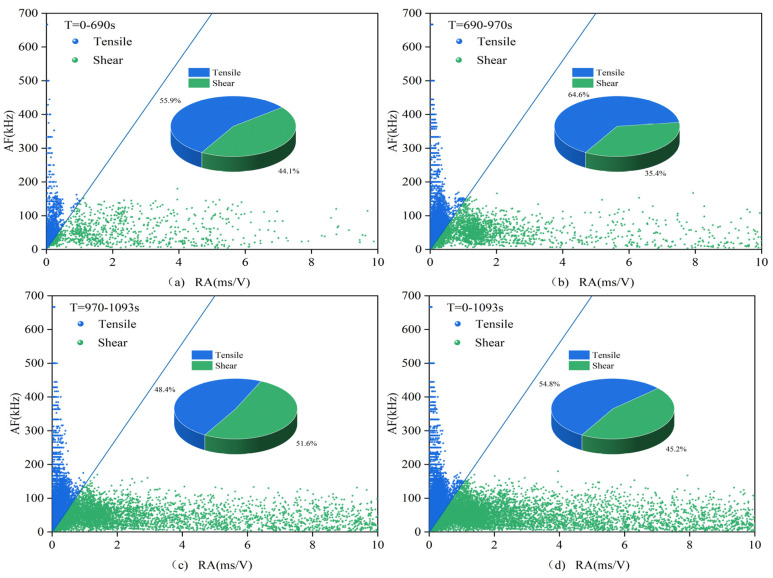
RA–AF scatter plot for the unfilled sample 0.7-3.

**Figure 21:**
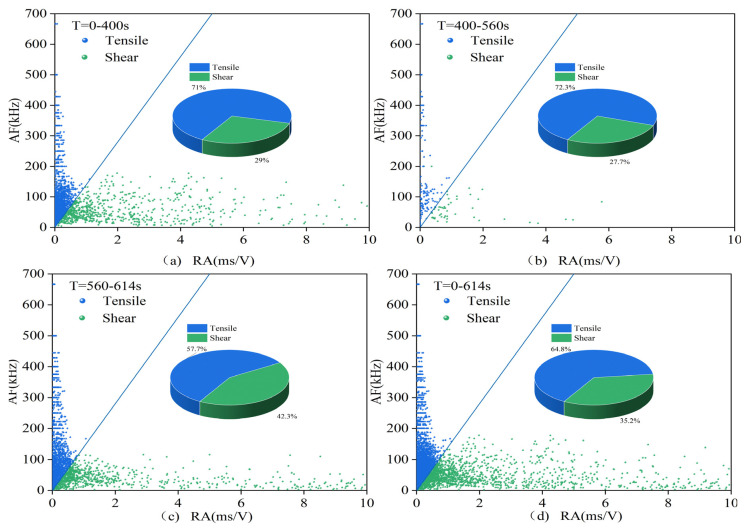
RA–AF scatter plot for the filled sample 2-1.

**Figure 22:**
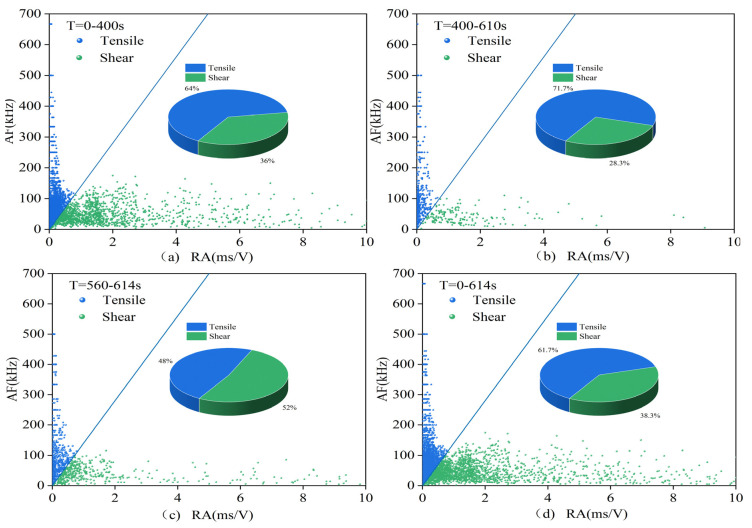
RA–AF scatter plot for the unfilled 2.0 mm group sample.

The acoustic emission data are roughly processed according to the compaction stage, crack development stage, failure stage, and post-failure zone, i.e., according to the first stage, the second and third stages, and the fourth and fifth stages in the stress–strain curve, as well as the sum of each stage situation. The data are processed according to the RA–AF index and signal partitioning, resulting in the signal proportions shown in Table 3 and the scatter distribution seen in Figure 19 and Figure 20, Table 4 corresponds to Figure 21 and Figure 22. In these figures, (**a**) represents the first stage, (**b**) represents the second and third stages, (**c**) represents the fourth and fifth stages, and (**d**) represents the overall situation of all stages.

(1) 0.7 mm and 1.0 mm

In the first three stages of all specimens, tensile signals dominate, and the difference in signal proportion between specimens with different crack widths is within 6%. However, from the fourth stage to the fifth stage, namely, the failure stage and the post-failure zone, the proportions of tensile signals show significant differences. Filled specimens mainly exhibit tensile cracks, while unfilled specimens are dominated by shear cracks. After MICP filling, the proportion of tensile signals in the sample significantly increases, indicating that MICP filling can effectively reduce the proportion of shear signals and suppress the generation and development of shear cracks. Therefore, the failure mode of the specimens shifts from being dominated by shear failure to being dominated by tensile failure, with vertical cracks being the primary manifestation.

(2) 1.5 mm group and 2.0 mm group

In the 1.5 mm group, the overall proportion of tensile signals in the unfilled specimens is even greater than that in the filled specimens. However, in the second and third stages, the proportion of tensile signals in the filled specimens is still higher, indicating that the mitigating effect of the filling material is mainly reflected in the second and third stages, with insufficient residual effects in the fourth stage. The small difference between the two in the failure stage is related to the instability of the specimens caused by the presence of cracks. MICP filling makes the failure process of the specimens more stable but has a relatively minor impact on the failure process. The root cause lies in the partial damage in the second and third stages, which leads to poor residual effects.

In the 2.0 mm group, as shown in the figures, the proportion of tensile signals in the first three stages is relatively high, and even higher than that in the 0.7 and 1.0 mm group specimens. However, it decreases in the failure stage, and the overall failure mode is dominated by tensile failure. The overall proportion of tensile signals in the specimens is higher than those in the first two groups. There is no significant difference between the two types of specimens, and the effect of MICP filling is not evident. The filling partially breaks in the first stage, leading to a more advanced failure time compared to the 1.5 mm group, resulting in worse residual effects, which is consistent with the results of acoustic emission events, amplitude, count, energy, and other parameters.

### 5.2. Comparison of Experimental Results

The RA–AF results indicate that in the 0.7 and 1.0 mm group, the proportion of tensile signals in most specimens is approximately 55–65%, suggesting that tensile failure is predominant in the experiments. The proportion of tensile signals remains relatively stable during the failure stage of the specimens. In the filled specimens, the proportion of tensile signals is higher than that in the unfilled specimens, indicating that while the overall failure is reduced after filling, the risk of shear failure is also decreased. In the 1.5 mm group, the filling material is damaged during the second and third stages without affecting the failure stage of the rock. In the 2.0 mm group, partial damage even occurs in the first stage, with no significant difference between the two types of specimens, resulting in unsatisfactory filling effects.

**Figure 23:**
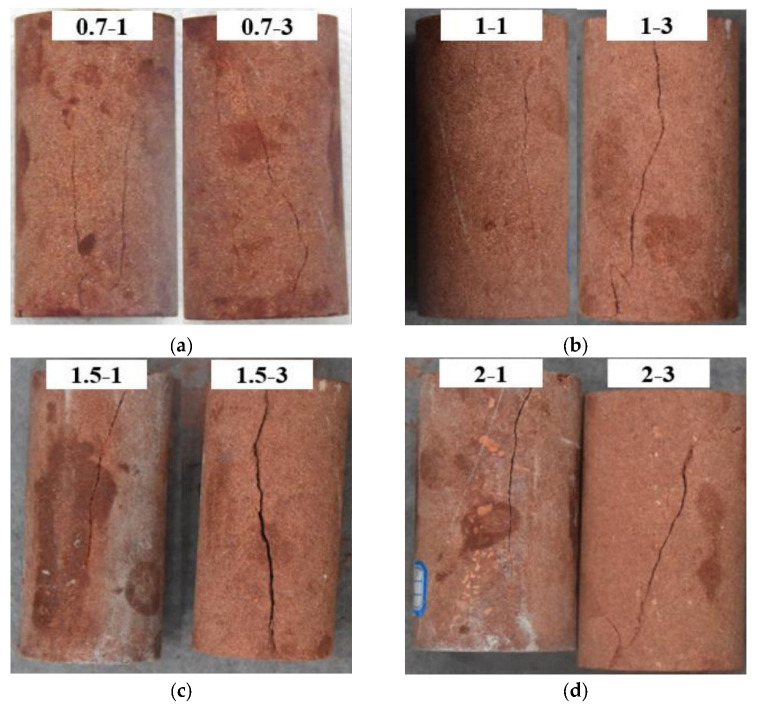
Macroscopic fracture morphology of the specimens. (**a**) 0.7 mm (**b**) 1.0 mm (**c**) 1.5 mm (**d**) 2.0 mm.

Consistent with the experimental findings, the results show that both types of specimens produce a brief burst of noise when they become unstable, in line with the failure characteristics of most brittle materials. As shown in Figure 23, the cracks in the specimens can be roughly classified into three types: one is tensile failure with cracks parallel to the axial direction, another is shear failure with cracks at a certain angle to the axial direction, and the third is a combination of both types, forming composite cracks. The crack patterns are consistent with the post-uniaxial compression fracture state of the specimens. As seen in Figure 23a,b, the 0.7 mm and 1 mm crack-width specimens remain relatively intact after the experiment. At the macroscopic level, the surface of the filled crack specimens presents a relatively straight crack that penetrates the surface, characterized by tensile failure and fewer cracks. The surface of the unfilled specimens also features a diagonal crack that penetrates the surface, with the crack forming an angle of approximately 30° with the vertical direction. Simultaneously, some short vertical cracks are distributed, still dominated by tensile failure but with a lower shear signal proportion in the unfilled specimens. In the filled specimens of the 0.7 and 1.0 groups, the cracks are parallel to the axial direction, while in the 1.0 group, they are mostly shear or composite cracks, consistent with the tensile/shear signal ratio obtained from RA–AF signals. In the 1.5 group, the cracks in the unfilled specimens are even simpler than those in the filled specimens, being mainly characterized by tensile cracks, which is also consistent with the RA–AF signals. In the 2.0 mm group, the crack in the filled specimen 2-1 is relatively vertical, while the crack in the unfilled specimen 2-3 is a composite crack, comprising both tensile and shear cracks. Overall, the RA–AF signal results show good consistency with the actual crack patterns observed in the specimens.

### 5.3. Discussion of Results

In this experiment, the results were compared with those of Liu X.L. [43], Wang G.L. [17], Li J.S. [44], and Dong L.J. [45]. Liu X.L. found that when the loading rate was greater than 3.0 × 10^−4^, the tensile signal of intact rock accounted for a proportion close to that of the shear signal, while at 2.5 × 10^−3^, the proportion of the tensile signal exceeded that of the shear signal. This indicates that the tensile signal should dominate when intact rock is broken, which is consistent with the results of this experiment at a loading rate of 1.0 × 10^−3^. Wang G.L. mentioned that in the failure of fractured rock, the proportion of shear signals during the failure stage was about 0.7, while in the failure process of intact rock, the proportion of tensile signals was also about 0.7. Before repair, this experiment was in good agreement with fractured rock, and after repair, it was close to intact rock but still unable to achieve the same effect, which is consistent with the facts. Li Jiashen also mentioned that under uniaxial compression loading conditions, the rupture source of intact red sandstone at the critical failure stage is mainly the tensile source, which is in good agreement with the results regarding the repaired rock in this paper.

Dong L.J. [45] found that the proportion of shear cracks gradually increased during the entire loading process of uniaxial compression. The results indicated that the increase of shear cracks is an important sign of unstable crack propagation and merging. This is also consistent with the results of the 1.5 mm and 2.0 mm specimens in this paper.

## 6. Conclusions

This study systematically investigated the fracture patterns, AE event rate, AE amplitude, AE count, and AE energy of MICP-filled rock specimens with prefabricated crack widths of 0.7–2.0 mm through uniaxial compression acoustic emission experiments, as well as the RA–AF variations. The main conclusions are as follows:

(1) As the crack width increases, the filling effect gradually decreases. The filling effect is significant at 0.7 mm and 1.0 mm, but it declines noticeably at 1.5 mm and is difficult to discern at 2.0 mm, which is related to the grouting effect.
(2) MICP filling significantly affects the failure process of sandstone. This is mainly manifested in the substantial occurrence of acoustic emission events at low-to-medium stress levels after filling, while the acoustic emission characteristics at high stress levels are similar to those of unfilled fractured sandstone. This is because the repaired portion has been mostly damaged at high stress levels, resulting in minimal residual effects.
(3) Using the amount of calcium carbonate generated as a single factor is not suitable for measuring the filling effect. Instead, a combination of indicators such as calcium carbonate filling rate, acoustic emission event rate, and other factors should be used to evaluate the MICP filling effect. There is a critical crack width in the filling process, beyond which the repair effect is poor or even difficult to discern.
(4) The failure process of rock is dominated by tensile signals, and the signal proportion varies in different stages. The proportion of tensile signals in the repaired specimens is higher, reducing the proportion of shear failure and altering the failure mode of the rock. The cracks become more parallel to the axial direction, and when the crack width is relatively small, the failure stage shifts from being dominated by shear signals to being dominated by tensile signals, which is consistent with the experimental results.

This passage examines the efficacy of utilizing microbiologically induced calcium carbonate precipitation (MICP) to mend cracks. The research indicates that the crack width plays a crucial role in the repair effectiveness and suggests the employment of multiple injections or alternative techniques for rock masses with crack widths exceeding the critical value. Furthermore, the study recommends novel evaluation indicators for the MICP repair effect, encompassing alterations in acoustic emission parameters and fracture patterns. The investigation also presents optimization strategies for enhancing MICP technology, such as broadening the scope of crack repair by incorporating large particle molecules into the slurry or utilizing multiple injections. However, the crack width was inadequate, and the critical repair width could not be precisely ascertained. In the subsequent step, new control groups of 1.0, 1.25, 1.5, 1.75, and 2.0 mm should be incorporated to determine the critical width and the width with a significant decrease in repair effectiveness. Microscopic analysis should be intensified, including the application of digital image correlation (DIC) technology and comparison with macroscopic analysis. Additionally, triaxial experiments should be conducted to investigate the performance and properties of rock repair under confining pressure.

## Figures and Tables

**Table 1:** Measurements of the physical properties of fractured sandstone before and after filling.

No.	Fracture Width (mm)	Filled or Not	Pre /g	After /g	Filled Mass /g	Dry Density /g·cm^3^	Pre-Filling Wave Speed	After-Filling Wave Speed	Compressive Strength (MPa)
0.7-1	0.7	Y	427.25	431.6	4.35	2.54	1428.57	1923.08	22.72
0.7-2	0.7	Y	423.06	427.51	4.55	2.51	1315.79	1666.67	30.01
0.7-3	0.7	N	422.41	422.41	/	2.51	1315.79	1315.79	20.12
0.7-4	0.7	N	416.05	416.05	/	2.47	1315.79	1315.79	29.8
1-1	1	Y	390.21	395.89	5.86	2.5	1111.11	1666.67	22.88
1-2	1	Y	419.21	424.7	5.49	2.68	1162.79	1666.67	27.45
1-3	1	N	417.66	417.66	/	2.67	1315.79	1315.79	26.77
1-4	1	N	422.8	422.8	/	2.71	1315.79	1315.79	32.53
1.5-1	1.5	Y	409.84	419.35	9.51	3.01	1282.05	1922.47	19.52
1.5-2	1.5	Y	407.48	414.35	6.87	2.99	1250	1724.14	25.53
1.5-3	1.5	N	385.76	385.76	/	2.83	1219.51	1219.51	22.99
1.5-4	1.5	N	409.91	409.91	/	3.01	1351.35	1351.35	28.21
2-1	2	Y	411.73	421.66	9.93	3.54	1219.51	1924.18	27.18
2-2	2	Y	377.93	387.3	9.37	3.25	1219.51	1724.14	20.21
2-3	2	N	420.66	420.66	/	3.62	1315.79	1315.79	41.36
2-4	2	N	407.53	407.53	/	3.51	1250	1250	30.69

**Table 2:** Red sandstone chemical composition contents.

Components	Content %
SiO_2_	58.2
CaCo_3_	15.8
NaAISi_3_O_8_	13.5
K_2_O	12.5

**Table 3:** Proportion of RA–AF tensile signals for the 0.7 and 1.0 group.

	a	b	c	d
0.7-1	51.7%	69.4%	65.5%	59.8%
0.7-3	55.9%	64.6%	48.4%	54.8%
1-1	59.4%	66.4%	53.5%	58.7%
1-3	54.8%	65.7%	47.7%	54.5%

**Table 4:** Proportion of RA–AF tensile signals for the 1.5 and 2.0 group.

	a	b	c	d
1.5-1	55.5%	63.2%	51.3%	54.8%
1.5-3	68.1%	60.5%	67.0%	63.0%
2-1	71.0%	72.3%	57.7%	64.8%
2-3	64.0%	71.7%	48.0%	61.7%

## Data Availability

The data that support the findings of this study are available upon request from the authors.
